# Homeostatic plasticity shapes the visual system’s first synapse

**DOI:** 10.1038/s41467-017-01332-7

**Published:** 2017-10-31

**Authors:** Robert E. Johnson, Nai-Wen Tien, Ning Shen, James T. Pearson, Florentina Soto, Daniel Kerschensteiner

**Affiliations:** 1 Department of Ophthalmology and Visual Sciences, Washington University School of Medicine, Saint Louis, MO 63110 USA; 2 Graduate Program in Neuroscience, Washington University School of Medicine, Saint Louis, MO 63110 USA; 3 Graduate Program in Developmental, Regenerative and Stem Cell Biology, Washington University School of Medicine, Saint Louis, MO 63110 USA; 4 Department of Neuroscience, Washington University School of Medicine, Saint Louis, MO 63110 USA; 5 Department of Biomedical Engineering, Washington University School of Medicine, Saint Louis, MO 63110 USA; 6 Hope Center for Neurological Disorders, Washington University School of Medicine, Saint Louis, MO 63110 USA

## Abstract

Vision in dim light depends on synapses between rods and rod bipolar cells (RBCs). Here, we find that these synapses exist in multiple configurations, in which single release sites of rods are apposed by one to three postsynaptic densities (PSDs). Single RBCs often form multiple PSDs with one rod; and neighboring RBCs share ~13% of their inputs. Rod-RBC synapses develop while ~7% of RBCs undergo programmed cell death (PCD). Although PCD is common throughout the nervous system, its influences on circuit development and function are not well understood. We generate mice in which ~53 and ~93% of RBCs, respectively, are removed during development. In these mice, dendrites of the remaining RBCs expand in graded fashion independent of light-evoked input. As RBC dendrites expand, they form fewer multi-PSD contacts with rods. Electrophysiological recordings indicate that this homeostatic co-regulation of neurite and synapse development preserves retinal function in dim light.

## Abstract (web summary)

Retinal rod bipolar cells (RBCs) partially undergo programmed cell death triggering cell density-dependent plasticity. This study shows that increased removal of RBCs using genetic approaches causes dendrites of the remaining RBCs to expand and contact more rod photoreceptors while reducing connectivity with each.

## Introduction

The ability of mammals to see in low light depends on the synapses between rods and rod bipolar cells (RBCs)^1^. Mutations in genes involved in the formation and function of these synapses cause congenital stationary night blindness (CSNB) in people^2^. Key molecular events in rod-RBC synapse assembly have been uncovered using mouse models of CSNB and other strategies^3–7^. A recent electron microscopy study showed that the spherical rod axon terminals (i.e., rod spherules) connect to varying numbers of RBC dendrites^8^, suggesting that rod-RBC synapse configurations might be malleable within molecularly defined boundaries. However, because only a few RBCs were reconstructed^8^, the range of configurations of rod-RBC synapses remains uncertain, and whether plasticity controls their distribution has not been tested.

Developmental plasticity is essential for the emergence of precise circuits; and its dysregulation underlies common neurodevelopmental disorders^9, 10^. Known plasticity mechanisms include axon and dendrite remodeling^11–13^, synapse formation and elimination^14–18^, and changes in the geometry and molecular architecture of synapses^19–21^. In developing circuits, populations of same-type neurons need to coordinate their connectivity to homogeneously cover input and target cell types, while individual neurons need to adjust their connectivity to avoid saturation and quiescence. Because most studies so far have focused on individual plasticity mechanisms and their underlying signals^22–24^, how different plasticity mechanisms (e.g., neurite remodeling and synapse formation) are co-regulated during development to optimize wiring of neuronal populations and individuals in vivo is unknown.

Throughout the developing nervous system, many neurons undergo programmed cell death (PCD), adjusting the complement and density of neuronal populations in emerging circuits^25, 26^. PCD triggers plasticity in the remaining neurons, which take over innervation of vacated inputs and targets. The retina is an ideal system for studying cell density-dependent plasticity, because axons and dendrites of each cell type cover synaptic layers uniformly^27, 28^. Cell density-dependent plasticity has been shown to regulate axon and dendrite growth of some retinal neurons^29, 30^ but not others^31, 32^. To what extent RBC axons and dendrites undergo cell density-dependent plasticity is incompletely understood^33^, and how cell density-dependent plasticity regulates synaptic development of any neuron is unknown.

To analyze the influence of cell density-dependent plasticity on RBC development and retinal circuit function, we generated mice in which ~53 and ~93% of RBCs, respectively, are removed by transgenic expression of diphtheria toxin concurrent with naturally occurring PCD^26^. We find that dendritic and axonal territories of the remaining RBCs increase in graded fashion to improve population coverage, whereas multi-PSD synapses on dendrites and synapse density of axons are reduced to restrain connectivity of individual RBCs. This coordinated plasticity of neurites and synapses occurs independent of light-evoked input from rods and preserves retinal output in dim light.

## Results

### Rod-RBC synapses exist in different configurations

**Fig. 1:**
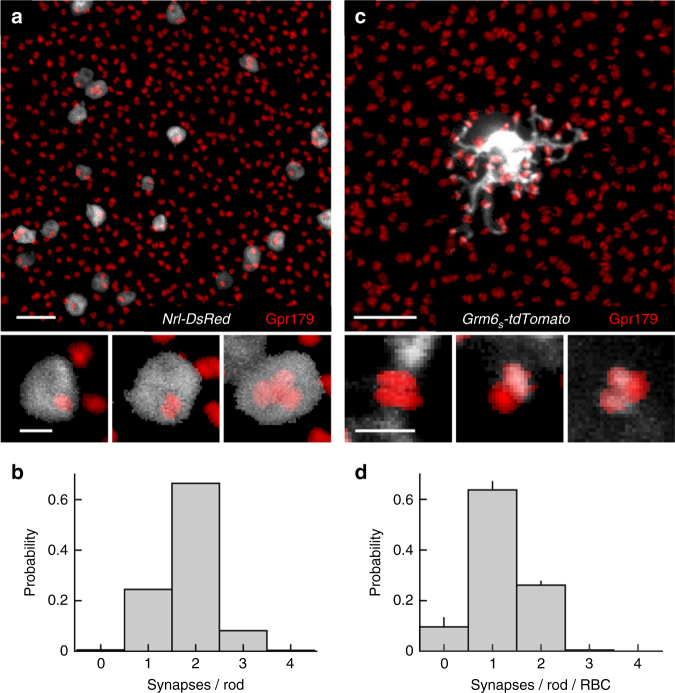
Rod-RBC synapses exist in different configurations. **a** Maximum intensity projection of a confocal image stack of the outer plexiform layer of a retina, in which rods were electroporated with *Nrl-DsRed* (gray) and in which RBC postsynaptic sites were stained for Gpr179 (red). Scale bar represents 5 μm. The top panel shows an overview, whereas the bottom panels present higher magnification views of individual rod spherules. **b** Histogram of the number of Gpr179 clusters per rod observed in our data (*n* = 555 rods, *n* = 12 mice). Scale bar represents 0.5 μm. **c** Analogous to **a**, but showing AAV-mediated (*Grm6*_*S*_ *-tdTomato*) labeling of an individual RBC in gray. For visual clarity, the RBC and rod synapses were digitally isolated in Amira. **d** Population data (mean ± SEM) showing the distribution of postsynaptic densities assembled by each RBC per rod spherule (*n* = 29 RBCs, *n* = 8 mice)

To examine the configurations of rod-RBC synapses, we first sparsely and selectively labeled rods by in vivo electroporation of a plasmid in which the fluorescent protein DsRed is expressed from promoter elements of the rod-specific neural retina leucine zipper (Nrl) transcription factor (Fig. 1a, *Nrl-DsRed*)^34, 35^. Each rod contains a single presynaptic ribbon^36, 37^. By contrast, we observed a range of RBC PSDs containing the probable G-protein coupled receptor 179 (Gpr179)^38–40^ in individual rod spherules (from one to three receptor clusters in >99% of spherules) (Fig. 1a, b). The distribution of RBC postsynaptic specializations per rod was similar when we stained for the metabotropic glutamate receptor mGluR6^3, 41^ instead of Gpr179, and when super-resolution rather than conventional confocal microscopy was used (Supplementary Fig. 1). We next explored how individual RBCs connect with rods in their dendritic fields. We generated adeno-associated viruses (AAVs) that expressed the fluorescent protein tdTomato from promoter elements of the *Grm6* gene, which encodes mGluR6 (*Grm6*_*S*_ *-tdTomato*)^42^. Intravitreal injections of *Grm6*_*S*_ *-tdTomato* labeled ON bipolar cells, which include RBCs and ON cone bipolar cells. RBCs could easily be identified by their characteristic morphology^15, 43^. We flat-mounted retinas of mice injected with *Grm6*_*S*_ *-tdTomato* and stained synaptic contacts for Gpr179 (Fig. 1c). Rod labeling had shown that overlapping Gpr179 clusters were invariably localized within the same spherule (Fig. 1a). We therefore counted overlapping Gpr179 clusters as synapses with a single rod, and determined whether a given cluster co-localized with a dendritic tip of the labeled RBC. We found that on average RBCs fail to be innervated ~10% of rods in their dendritic fields, assemble a single PSD in ~63% of spherules, and form multi-PSD contacts with ~27% of rods (Fig. 1c, d). Rod-RBC synapses thus exist in different configurations, in which a single presynaptic release site is apposed by one to three PSDs belonging to one or more RBCs.

### Dendrites of neighboring RBCs overlap and share rod input

**Fig. 2:**
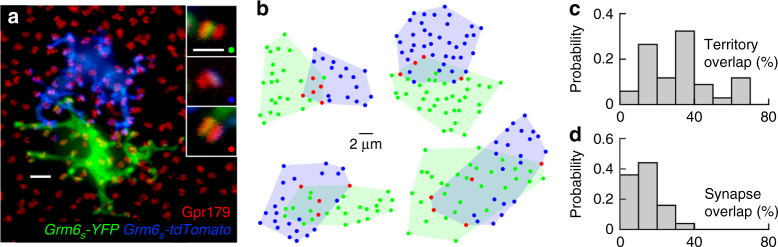
RBC dendrites overlap and share rod input. **a** Maximum intensity projection of a confocal image stack of two adjacent RBCs labeled with spectrally separable fluorophores via AAVs (*Grm6*_*S*_ *-tdTomato* in blue and *Grm6*_*S*_ *-YFP* in green). RBC postsynaptic sites are stained for Gpr179 (red). For visual clarity, the two RBCs were digitally isolated in Amira. Scale bar represents 2 μm. Insets on the right show higher magnification views of Gpr179 clusters contacted by either (top two panels) or both (bottom panel) RBCs. Scale bar represents 1 μm. **b** Representative examples of dendritic and synaptic overlap of four pairs of RBCs. Shaded areas represent dendritic territories. Rods targeted by dendrites of either RBC are marked by green and blue circles; rods targeted by dendrites of both RBCs are indicated by red circles. **c**, **d** Distributions of dendritic territory overlap (**c**, *n* = 37 pairs, *n* = 10 mice) and synaptic overlap (**d**, *n* = 28 pairs, *n* = 8 mice) between neighboring RBCs

To visualize dendritic interactions of neighboring RBCs we labeled cells with spectrally separable fluorescent proteins via two AAVs (*Grm6*_*S*_ *-tdTomato* and *Grm6*_*S*_ *-YFP*) and stained retinas for Gpr179 (Fig. 2a). We restricted our analysis to RBC pairs whose somata were <10 μm apart (center–center distance). Dendritic territories of these RBC pairs, defined as the smallest convex polygons encompassing all Gpr179-bearing dendritic tips, overlapped on average by 30%, with relatively large variability in the amount of overlap between pairs (coefficient of variation: 62%; Fig. 2b, c). By comparison, RBC pairs shared a smaller (13%) fraction of input from rods, and the variability in the fraction of input shared between RBC pairs was lower (coefficient of variation: 50%; Fig. 2b, d) than that of their dendritic overlap.

### Transgenic removal of RBCs from developing circuits

**Fig. 3:**
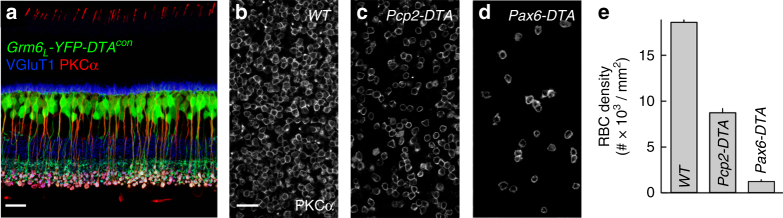
Transgenic removal of RBCs from developing circuits. **a** Section through a *Grm6L-YFP-DTA* ^*con*^ retina in which ON bipolar cells express YFP (green) stained for VGluT1 (blue), which labels photoreceptor and bipolar cell axon terminals, and for the RBC-specific marker PKCα (red). Scale bar indicates 20 μm. **b**–**d** Retinal flat mounts from wild-type (**b**), *Pcp2-DTA* (**c**), and *Pax6-DTA* (**d**) mice stained for PKCα. Scale bar indicates 20 μm. **e** Summary data (mean ± SEM) of RBC densities in wild-type (*n* = 23 mice), *Pcp2-DTA* (*n* = 8 mice), and *Pax6-DTA* (*n* = 22 mice) mice. By Kruskal–Wallis one-way ANOVA testing, the density of RBCs was lower in *Pcp2-DTA* and *Pax6-DTA* compared to wild-type retinas (*p* < 0.04 and *p* < 10^−8^, respectively), and lower in Pax6-DTA than in Pcp2-DTA retinas (*p* < 0.049)

To probe the influence of cell density-dependent plasticity on neurite and synapse development, we generated mice that conditionally express an attenuated version of diphtheria toxin^44^ in RBCs and ON cone bipolar cells (*Grm6*_*L*_ *-YFP-DTA* ^*con*^ mice^16^, Fig. 3a). We crossed *Grm6*_*L*_ *-YFP-DTA* ^*con*^ mice to *Pcp2-Cre* ^45^ or *Pax6-Cre* ^46^ lines to produce *Pcp2-DTA* and *Pax6-DTA* mice, respectively. In *Pcp2-Cre* mice, Cre recombinase is expressed in ~50% of RBCs and in a small subset of photoreceptors (Supplementary Fig. 2a–d). Accordingly, cell removal in *Pcp2-DTA* mice is restricted to RBCs, whose density is reduced by ~53% (Fig. 3b, c, e). In *Pax6-Cre* mice, Cre is expressed in retinal progenitor cells and recombination therefore occurs in all retinal cell types. Cre expression in these mice is region specific and excludes a dorsoventral wedge through the center of the retina (Supplementary Fig. 2e)^46^. In the Cre-positive regions of *Pax6-DTA* retinas, ~93% of RBCs are removed without microglial activation (Fig. 3b, d, e; Supplementary Fig. 3). In addition to RBCs, ON cone bipolar cells in the Cre-positive regions are deleted to varying degrees^47^. By staining for the cell type-specific marker PKCα at different postnatal ages, we found that RBCs in *Pax6-DTA* mice are removed around postnatal day 9 (P9: 80% < WT, P15: 93% < WT). Thus, we have generated mice (*Pcp2-DTA* and *Pax6-DTA*), in which distinct fractions of RBCs are removed from circuits concurrent with developmental PCD^26^.

### Cell density regulates RBC dendrite and synapse development

**Fig. 4:**
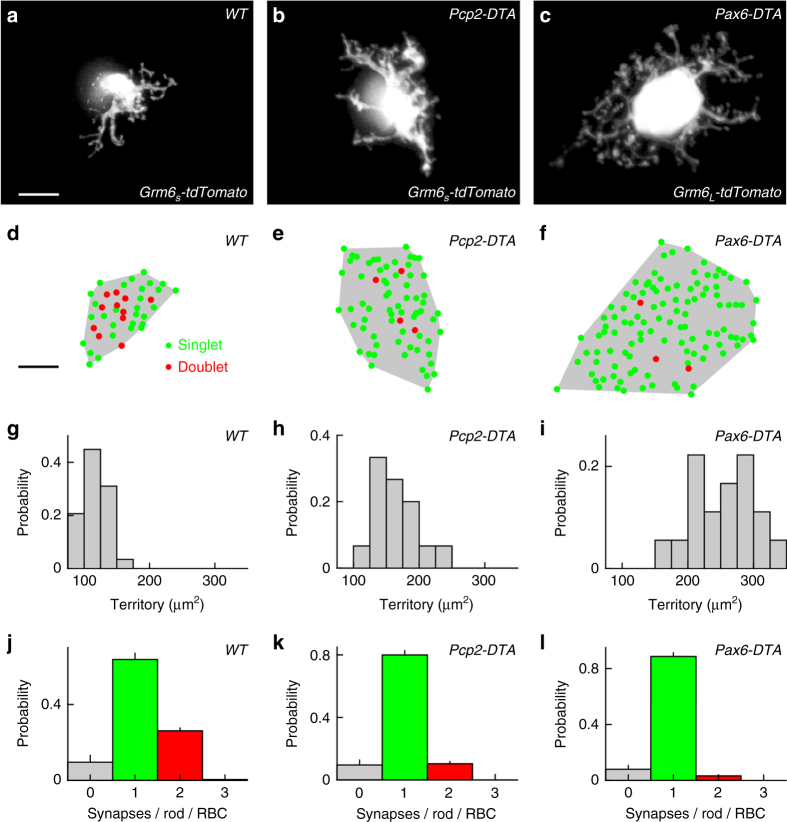
Cell density regulates RBC dendrite and synapse development. **a**–**c** Maximum intensity projection of dendritic trees of individual RBCs labeled via AAVs (*Grm6*_*S*_ *-tdTomato*) or in a transgenic line (*Grm6*_*L*_-*tdTomato*) in wild-type (**a**), *Pcp2-DTA* (**b**), and *Pax6-DTA* (**c**) mice. For visual clarity, RBCs were digitally isolated in Amira. Scale bar indicates 5 μm. **d**–**f** Maps of dendritic territories (gray shaded areas) and synapse configurations (singlets: green circles, doublets: red circles) of the cells shown in **a**–**c**. Scale bar indicates 5 μm. **g**–**i** Summary data of RBC dendritic territories in wild-type (**g**, *n* = 29 RBCs, *n* = 8 mice), *Pcp2-DTA* (**h**, *n* = 15 RBCs, *n* = 6 mice), and *Pax6-DTA* (**i**, *n* = 18 RBCs, *n* = 6 mice) mice. By Kruskal–Wallis one-way ANOVA testing, RBC dendrite territories in *Pcp2-DTA* and *Pax6-DTA* retinas were larger than in wild-type retinas (*p* < 0.005 and *p* < 10^−8^, respectively), and RBC dendrite territories were larger in *Pax6-DTA* than in *Pcp2-DTA* retinas (*p* < 0.02). **j**–**l** Population data (mean ± SEM) of the distribution of synapses configurations on RBC dendrites in wild-type (**j**, *n* = 29 RBCs, *n* = 8 mice), *Pcp2-DTA* (**k**, *n* = 15 RBCs, *n* = 6 mice), and *Pax6-DTA* (**l**, *n* = 10 RBCs, *n* = 4 mice) retinas. By Kruskal–Wallis one-way ANOVA testing, the average number of PSDs per rod and RBC was lower in *Pax6-DTA* than in wild-type retinas (*p* < 0.003)

We used *Pcp2-DTA* and *Pax6-DTA* mice to study the influence of cell density-dependent plasticity on RBC dendrites and rod-RBC synapses. RBCs were labeled using either a transgenic line (*Grm6*_*L*_-*tdTomato*) or AAVs (*Grm6*_*S*_-*tdTomato*). RBC morphologies were indistinguishable between these labeling strategies and results from both approaches were therefore combined. Comparisons of wild-type, *Pcp2-DTA*, and *Pax6-DTA* retinas revealed that RBC dendrites expand in graded fashion as the density of RBCs is reduced (Fig. 4a–i). To determine whether dendrite growth is regulated by local (e.g., contact-mediated) or global (e.g., long-range diffusible messenger) signals, we analyzed the morphology of RBCs at the border of Cre-positive and Cre-negative regions in *Pax6-DTA* retinas. There, we frequently observed clusters of RBCs surrounded by RBC-depleted areas. The dendrites of RBCs in such clusters invariably extend away from remaining neighbors into the depleted areas (Supplementary Fig. 4). This suggests that, similar to other retinal neurons^29, 30^, the growth of RBC dendrites is constrained by local homotypic signals.

To test whether local interactions are required to maintain RBC dendrite size in the adult retina, we crossed *Pcp2-Cre* mice to a transgenic strain in which the diphtheria toxin receptor is expressed in a Cre-dependent manner (*DTR* mice)^48^. However, diphtheria toxin injections that completely remove other retinal cells targeted with this strategy^49, 50^ caused only a minor reduction in RBC density in double-positive offspring (*Pcp2-DTR*, Supplementary Fig. 5). We therefore could not analyze the extent of cell density-dependent plasticity in the adult retina.

The density of rods is unchanged in *Pcp2-DTA* and *Pax6-DTA* mice (Supplementary Fig. 6), and as RBC dendrites in their retinas expand, they contact an increasing number of rods (Fig. 4a–f). This improves input coverage by the remaining RBC population, but carries the risk of saturating input to individual cells. Interestingly, analysis of rod-RBC synapses revealed that whereas RBCs in wild-type retinas form two (i.e., doublets) or more PSDs with 27% of rods, the frequency of PSD doublets is gradually reduced in *Pcp2-DTA* (10%) and *Pax6-DTA* (3%) retinas. This homeostatic shift from doublet to singlet (i.e., one PSD with one rod spherule) synapses could serve to limit input to expanded dendrites.

### RBC plasticity is independent of light-evoked rod input

**Fig. 5:**
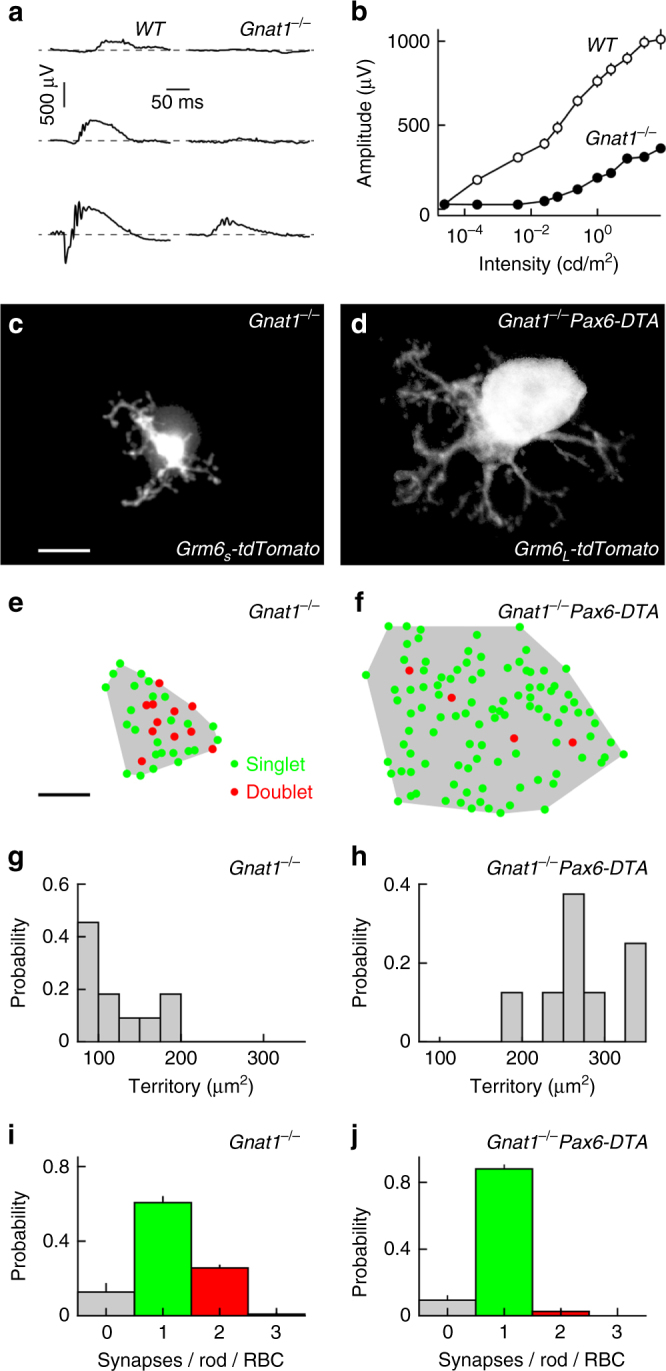
RBC plasticity is independent of light-evoked rod input. **a** Representative ERG responses to stimuli of increasing light intensity (top row: 0.00025 cd/m^2^, middle row: 0.25 cd/m^2^, bottom row: 25 cd/m^2^) recorded from wild-type (left column) and *Gnat1* ^*−/−*^ (right column) retinas. **b** Summary data of intensity response functions measured from b-wave amplitudes in wild-type (open circles, *n* = 6 mice) and *Gnat1* ^*−/−*^ (filled circles, *n* = 4 mice) animals. **c**, **d** MIP of dendritic trees of individual RBCs labeled via AAVs (*Grm6*_*S*_ *-tdTomato*) or in a transgenic line (*Grm6*_L_-*tdTomato*) in *Gnat1* ^*−/−*^ (**c**) and *Gnat1* ^*−/−*^ *Pax6-DTA* (**d**) mice. For visual clarity, RBCs were digitally isolated in Amira. Scale bar indicates 5 μm. **e**, **f** Maps of dendritic territories (gray shaded areas) and synapse configurations (singlets: green circles, doublets: red circles) of the cells shown in **c** and **d**. Scale bar indicates 5 μm. **g**, **h** Summary data of RBC dendritic territories in *Gnat1* ^*−/−*^ (**g**, *n* = 13 RBCs, *n* = 5 mice) and *Gnat1* ^*−/−*^ *Pax6-DTA* (**i**, *n* = 8 RBCs, *n* = 4 mice, *p* < 0.001) mice. **i**, **j** Population data (mean ± SEM) of the distribution of synapses configurations on RBC dendrites in *Gnat1* ^*−/−*^ (**i**, *n* = 11 RBCs) and *Gnat1* ^*−/−*^ *Pax6-DTA* (**j**, *n* = 8 RBCs, *n* = 4 mice, *p* < 0.04) retinas

We next tested to what extent the remodeling of dendrites and synapses elicited by changes in RBC density is regulated by input from rods. In mice lacking rod transducin-α (*Gnat1* ^*−/−*^ mice), rods fail to respond to light and scotopic ERG responses are suppressed (Fig. 5a, b)^51^. We found that RBC dendrites in *Gnat1* ^*−/−*^ mice occupy normal territories and develop synapses with configurations similar to those observed in wild-type retinas (Fig. 5c, e, g, i). Moreover, in *Gnat1* ^*−/−*^ *Pax6-DTA* mice, RBC dendrites expand and shift from doublet to singlet synapses as they do in *Pax6-DTA* mice (Fig. 5d, f, h, j). Thus, dendrite and synapse development, and cell density-dependent plasticity of RBCs appear to be independent of light-evoked input from rods.

To silence spontaneous transmitter release from rods, we crossed mice in which the light chain of tetanus toxin (TeNT) is expressed in a Cre-dependent manner (*TeNT* mice)^52^ to *Rhodopsin-iCre* mice, which express a codon-improved version of Cre recombinase (iCre)^53^, under control of the rod-specific rhodopsin promoter^54^. Unfortunately, double-positive offspring (*Rhodopsin-TeNT*) showed rapid photoreceptor degeneration (Supplementary Fig. 7). We were therefore unable to analyze the influence of spontaneous transmitter release from rods on RBC dendrite and synapse development and plasticity.

### Cell density regulates RBC axon and synapse development

**Fig. 6:**
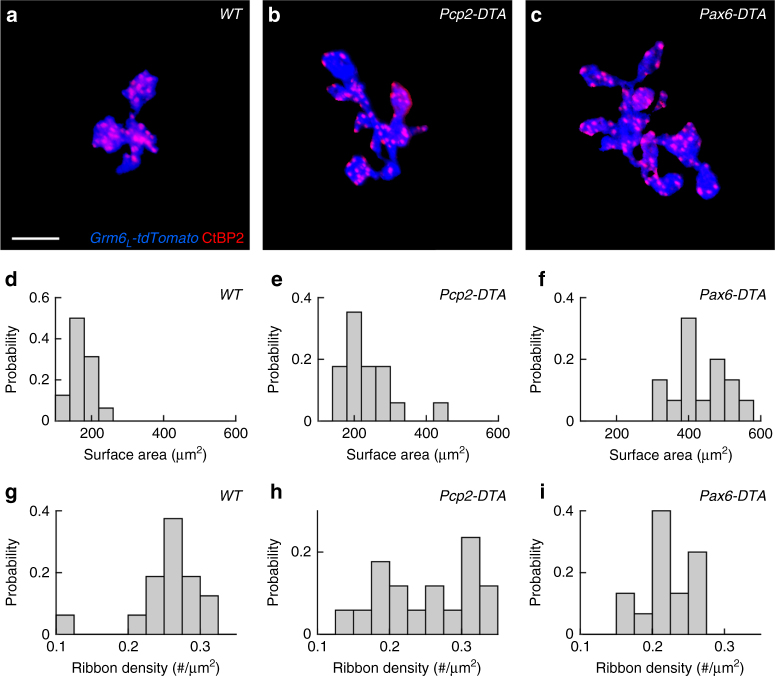
Cell density regulates RBC axon and synapse development. **a**–**c** Maximum intensity projection of RBC axon terminals labeled by transgenic expression of tdTomato (*Grm6*_L_ *-tdTomato* in blue) and presynaptic release sites stained for the C-terminal binding protein 2 (CtBP2, red) in wild-type (**a**), *Pcp2-DTA* (**b**), and *Pax6-DTA* (**c**) retinas. For visual clarity, RBC axons and their synapses were digitally isolated in Amira. Scale bar indicates 5 μm. **d**–**f** Summary data of RBC axon surface areas in wild-type (**d**, *n* = 16 RBCs), *Pcp2-DTA* (**e**, *n* = 17 RBCs), and *Pax6-DTA* (**f**, *n* = 15 RBCs) mice. By Kruskal–Wallis one-way ANOVA testing, RBC axon surface areas in *Pcp2-DTA* and *Pax6-DTA* retinas were greater than in wild-type retinas (*p* < 0.02 and *p* < 10^−8^, respectively), and RBC axon surface areas were greater in *Pax6-DTA* than in *Pcp2-DTA* retinas (*p* < 0.003). **g**–**i** Population data (mean ± SEM) from wild-type (**g**, *n* = 16 RBCs), *Pcp2-DTA* (**h**, *n* = 17 RBCs), and *Pax6-DTA* (**i**, *n* = 15 RBCs) retinas show that synapse density tends to decrease with increasing axon size. By Kruskal–Wallis one-way ANOVA testing, RBC synapse densities in *Pcp2-DTA* and *Pax6-DTA* retinas were lower than in wild-type retinas (*p* < 0.003 and *p* < 0.02, respectively)

Given the homeostatic plasticity of RBC dendrites and rod-RBC connections, we next tested whether cell density similarly co-regulates the development of RBC axons and their synapses. We found that RBC axons expand in graded fashion as the density of RBCs around them decreases in *Pcp2-DTA* and *Pax6-DTA* mice (Fig. 6a–f). Labeling for C-terminal binding protein 2, a component of presynaptic ribbons^55^, then showed that the density of RBC output synapses is reduced in *Pcp2-DTA* and even further in *Pax6-DTA* retinas (Fig. 6g–i). Thus, cell density-dependent plasticity co-regulates axon size and synapse density in seemingly homeostatic fashion, similar to the changes observed in RBC dendrites and rod-RBC synapse configurations.

### RBC plasticity preserves retinal output in dim light

**Fig. 7:**
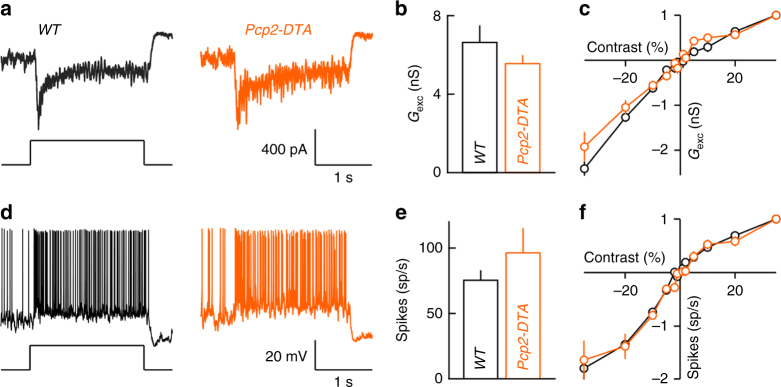
Excitatory input and spike responses of ONα-RGCs in wild-type and *Pcp2-DTA* mice. **a** EPSC responses to dim light steps (three rhodopsin isomerization/rod/s, 3 R*) recorded from ONα-RGCs in wild-type (left, black) and *Pcp2-DTA* (right, orange) retinas. **b** Summary data (mean ± SEM) of the excitatory conductances elicited by dim light steps (as shown in **a**) in ONα-RGC of wild-type (black, *n* = 7 cells, *n* = 2 mice) and *Pcp2-DTA* (orange, *n* = 6 cells, *n* = 2 mice, *p* > 0.5 by Wilcoxon rank sum test) mice. **c** Population data for contrast response functions of excitatory conductances of ONα-RGCs recorded in wild-type (black, *n* = 6 cells, *n* = 2 mice) and *Pcp2-DTA* (orange, *n* = 3 cells, *n* = 2 mice, *p* > 0.1 by bootstrapping methods) retinas. **d**–**f** Analogous to **a**–**c**, but for spike responses instead of excitatory inputs. Responses to dim light steps (wild type *n* = 9 cells, *n* = 2 mice *Pcp2-DTA n* = 5 cells, *n* = 2 mice, *p* > 0.3 by Wilcoxon rank sum test), and contrast response functions (wild type *n* = 6 cells, *n* = 2 mice, *Pcp2-DTA n* = 3 cells, *n* = 2 mice, *p* > 0.3 by bootstrapping methods) were not significantly different between wild-type and *Pcp2-DTA* mice

We hypothesized that the homeostatic co-regulation of neurites and synapses of RBC dendrites and axons serves to improve input and target coverage by the remaining population of RBCs, while maintaining manageable input and output connectivity for individual neurons; and that this in turn preserves retinal function in dim light. To test this hypothesis, we recorded the synaptic input and spike responses of ONα retinal ganglion cells (ONα-RGCs), which are sensitive to small changes in luminance (i.e., low contrast) even in dim light^56^. In Cre-positive regions of *Pax6-DTA* mice, few RBCs are left and varying fractions of cone bipolar cells are deleted. We therefore focused on *Pcp2-DTA* retinas, in which ~53% of RBCs are selectively removed. Whole-cell patch clamp recordings revealed that in spite of this loss, all ONα-RGCs (11 of 11 cells) responded to stimuli at light levels preferentially activating the rod bipolar pathway (Supplementary Fig. 8). Moreover, the amplitudes of excitatory inputs and spike responses of ONα-RGCs were unchanged in *Pcp2-DTA* compared to wild-type retinas (Fig. 7a, b, d, e), and the characteristically linear contrast response functions of ONα-RGCs were preserved in *Pcp2-DTA* mice (Fig. 7c, f).

**Fig. 8:**
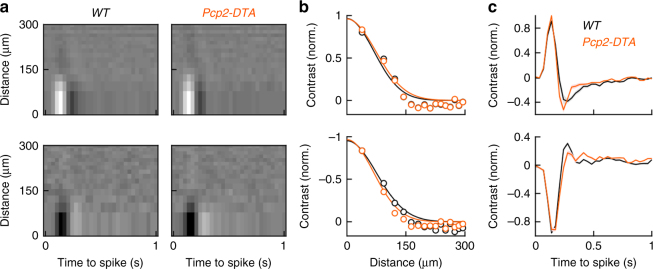
Spatiotemporal receptive fields of ONα-RGCs and OFFα-RGCs in wild-type and *Pcp2-DTA* mice. **a** Representative spatiotemporal receptive field maps from ONα-RGCs (top row) and OFFα-RGCs (bottom row) in wild-type and *Pcp2-DTA* mice. Because the area of each ring in our circular white noise stimuli was kept constant, rows in the receptive field maps decrease in height with increasing distance from the center. **b**, **c** Summary data (mean ± SEM) of the spatial and temporal response profiles at the temporal and spatial absolute response maxima, respectively (WT ONα-RGC *n* = 12 cells, WT OFFα-RGCs *n* = 9, *n* = 4 mice; *Pcp2-DTA* ONα-RGC *n* = 6, *Pcp2-DTA* OFFα-RGC *n* = 7, *n* = 3 mice)

RGC types differ in their spatiotemporal receptive fields. To test whether RGC type-specific receptive field properties are altered in *Pcp2-DTA* mice, we recorded spike trains of ONα-RGCs and OFFα-RGCs during presentation of circular white noise stimuli. In these stimuli, the intensities of rings of equal area centered on the recorded cell were chosen at random every 33 ms (refresh rate: 30 Hz) from a Gaussian distribution. We then mapped receptive fields by spike-triggered stimulus averaging^57, 58^. Receptive field maps of ONα-RGCs and OFFα-RGCs were indistinguishable between wild-type and *Pcp2-DTA* mice (Fig. 8a–c). Thus, in addition to contrast coding, cell type-specific spatiotemporal filtering of visual signals is preserved in *Pcp2-DTA* mice, supporting the notion that cell density-dependent plasticity co-regulates neurite and synapse development of RBCs to preserve retinal function in dim light.

## Discussion

A recent electron microscopy study reconstructed the connectivity patterns of eight RBCs in mice, and found that signals from one rod often diverge to multiple RBCs and that one RBC can form multiple synapses with a single rod^8^. Similar observations had previously been made in cat and primate retinas^59, 60^. Using in vivo electroporation and AAVs to label rods and RBCs, respectively, we analyzed the connectivity of a large number of these cells. Our findings confirm the diversity of rod-RBC synapse configurations, and give a more comprehensive account of their distribution (Fig. 1). In addition, we visualize dendritic interactions among neighboring RBCs. On average, we find that dendrites of adjacent RBCs overlap by ~30% and share ~13% of their input from rods (Fig. 2). Based on physiological evidence, Pang et al.^61^ suggested that the mouse retina may contain two distinct types of RBCs. Dendritic overlap and shared input among neighboring RBCs in our study form single continuous distributions (Fig. 2). Together with recent large-scale single cell expression profiling data^62^, this argues for a single RBC type, whose function may vary.

In *Pcp2-DTA* and *Pax6-DTA* mice, RBC dendrites expand in graded fashion (Fig. 4). Together with a previous study, which found an inverse relationship between RBC density and dendrite size across mouse strains^33^, our findings suggest that homotypic signals restrict dendrite growth of RBCs. At the border of Cre-negative and Cre-positive regions in *Pax6-DTA* retinas, we find that dendritic growth of RBCs is directed away from remaining neighbors, suggesting that homotypic signals are local, possibly mediated by cell–cell contacts (Supplementary Fig. 4). The cell adhesion molecule DSCAM-LIKE 1 (DSCAML1) mediates repulsive interactions between RBC dendrites^63^. However, while DSCAML1 is required for self-avoidance, dendrite size is reduced, rather than increased, in *Dscaml1* null mutants^63^. Thus, molecular identities of signals that control RBC dendrite size remain to be uncovered. The same or different signals may control RBC axon size, which increases in parallel with dendrite size in *Pcp2-DTA* and *Pax6-DTA* mice (Fig. 6). In principle, local imbalances in activity introduced by removal of a fraction of RBCs could contribute to the changes in axon size. We think this is unlikely, because studies that silenced subsets of cone bipolar cells found axon territories to be unchanged^14, 47^.

In addition to neurite territories, we find that cell density-dependent plasticity regulates synaptogenesis. As RBC dendrites expand in *Pcp2-DTA* and *Pax6-DTA* mice, they form fewer doublet and more singlet synapses with rods (Fig. 4). This shift in synapse configurations, in which the number of PSDs per presynaptic release site is adjusted, constitutes a novel plasticity mechanism. It is reminiscent of changes in multi-synaptic appositions in the inner retina^16^ and in same-dendrite multiple-synapse boutons in the hippocampus^64^. However, these architectures contain multiple presynaptic release sites, and were found to change during activity-dependent rather than cell density-dependent plasticity^16, 64^.

We find that RBC dendrite and synapse development, and cell density-dependent plasticity are unchanged in a *Gnat1* ^*−/−*^ background (Fig. 5). Similarly, a recent study found that clustering of mGluR6 receptors on RBC dendrites is not affected by dark rearing^37^. By contrast, rod-RBC synapses fail to form when vesicle fusion of rods is blocked by transgenic expression of tetanus toxin^3^. Thus, spontaneous rather than light-evoked signals from rods appear to shape the development of the rod bipolar pathway.

How different plasticity mechanisms of developing neurons are coordinated and to what end is not well understood. For RBC dendrites and axons in *Pcp2-DTA* and *Pax6-DTA* retinas, we find that adjustments of neurite territories are countered by opposite changes in synaptogenesis (Figs. 4, 6). Similar co-regulation of dendrite and synapse development was previously observed in Drosophila following perturbations of input activity^65^, indicating that coordinated plasticity may be an evolutionarily conserved feature of neural circuits. We propose that homeostatic co-regulation of neurite growth and synaptogenesis serves to simultaneously optimize wiring of neuronal populations (i.e., input and target coverage) and individuals (i.e., input and output connectivity). In patch clamp recordings, we find that dim light responses of ONα-RGCs and OFFα-RGCs in *Pcp2-DTA* mice are preserved, in spite of the ~53% reduction in RBCs (Figs. 7, 8). This highlights the ability of homeostatically co-regulated plasticity mechanisms to stabilize circuit function.

## Methods

### Mice

We generated mice in which a 9 kb fragment of the *Grm6* promoter drives expression of YFP or, upon Cre-mediated recombination, of an attenuated version of diphtheria toxin (*Grm6*_*L*_ *-YFP-DTA* ^*con*^)^16, 47^. To remove different numbers of RBCs from the developing retina, *Grm6*_*L*_ *-YFP-DTA* ^*con*^ mice were crossed to *Pax6-Cre* ^46^ (RRID:MGI:4821787) and *Pcp2-Cre* ^45^ (RRID: IMSR_JAX:010536) mice. We refer to double transgenic offspring from these crosses as *Pax6-DTA* and *Pcp2-DTA* mice, respectively. We tried to remove RBCs from the mature retina by crossing *Pcp2-Cre* mice, to a strain in which the diphtheria toxin receptor is expressed in a Cre-dependent manner (*DTR* mice, RRID:IMSR_JAX:007900)^48^. Double transgenic offspring from this cross (*Pcp2-DTR*) was injected with diphtheria toxin (1 μg/50 g body weight) intraperitoneally once every other day for a total of 4 days starting at P30^49^. To evaluate the effect of light-evoked signals from rod photoreceptors on synaptic wiring and plasticity, *Pax6-DTA* mice were crossed to mice lacking rod transducin-α (*Gnat1* ^*−/−*^ mice)^51^. To block neurotransmitter release from rod photoreceptors, we crossed *Rhodopsin-iCre* ^54^ mice to a strain in which the light chain of tetanus toxin is expressed in a Cre-dependent manner (*TeNT* mice)^52^. In a subset of experiments, RBCs were labeled transgenically (*Grm6*_*L*_ *-tdTomato*)^15^. All mice were crossed onto a C57BL/6J background for more than five generations. Experiments were conducted using young adult mice (postnatal day 25 (P25)–P35) of both sexes. Mice were kept on a 12 h light/12 h dark cycle. For anatomy experiments, mice were typically killed in the morning after 2-4 h of light. For electrophysiology experiments, mice were dark-adapted overnight and killed in the subjective morning. The procedures in this study were approved by the Animal Studies Committee of Washington University School of Medicine and performed in compliance with the National Institutes of Health Guide for the Care and Use of Laboratory Animals.

### Adeno-associated viruses

To label RBCs, we generated AAVs in which four concatenated repeats of a 200 bp fragment of the *Grm6* promoter^42^ drive expression of red (*Grm6*_*S*_ *-tdTomato*) or yellow fluorescent proteins (*Grm6*_*S*_ *-YFP*). The *pAAV-Grm6*_*S*_ *-YFP* plasmid was created by switching the *CAG* promoter of *pAAV-CAG-YFP* ^5^ with the *Grm6* repeats using linkers containing *Asp*718I and *Eco*RI restriction sites introduced by PCR. *pAAV-Grm6*_*S*_ *-tdTomato* was then derived from *pAAV-Grm6*_*S*_ *-YFP* by replacing YFP with tdTomato from a *tdTomato-N1* vector (Addgene #54642) using *Bam*HI and *Not*I restriction sites. AAV1/2 chimeric virions were produced by co-transfecting HEK-293 cells with *pAAV-Grm6*_*S*_ *-YFP* or *pAAV-Grm6*_*S*_ *-tdTomato*, and helper plasmids encoding Rep2 and the Cap for serotype 1 and Rep2 and the Cap for serotype 2. Forty-eight hours after transfection, cells and supernatant were harvested and viral particles purified using heparin affinity columns (Sigma). Viruses (250 nL) were delivered into the vitreous chamber of newborn mice anesthetized on ice via a Nanoject II injector (Drummond).

### In vivo electroporation

To label rod photoreceptors, we injected *pNrl-DsRed* plasmid^34^ into the subretinal space of newborn mice anesthetized on ice via a Nanoject II injector (Drummond). To electroporate rods, five 80 V square pulses of 50 ms duration generated by an ECM830 (BTX Harvard Apparatus) were delivered via tweezer electrodes with the anode placed on the injected eye^66^.

### Optic nerve crush

The optic nerve was exposed intraorbitally and crushed with forceps (Dumont #55 FST) for ~5 s ~1 mm behind the posterior surface of the eyeball.

### Tissue preparation

Mice were killed with CO_2_ and enucleated. For vibratome sections, the cornea, lens, and vitreous were removed in in HEPES-buffered mouse artificial cerebrospinal fluid (mACSF_HEPES_)—containing (in mM) 119 NaCl, 2.5 KCl, 2.5 CaCl_2_, 1.3 MgCl_2_, 1 NaH_2_PO_4_, 11 glucose, and 20 HEPES (pH adjusted to 7.37 with NaOH)—and the remaining eye cup fixed for 30 min in 4% paraformaldehyde in mACSF_HEPES_. For flat mount preparations, retinas were isolated in mACSF_HEPES_, mounted on membrane disks (HABGO1300, Millipore) and fixed for 30 min in 4% paraformaldehyde in mACSF_HEPES_. For electrophysiology, retinas from dark-adapted mice (>2 h) were isolated under infrared illumination in bicarbonate-buffered mouse artificial cerebrospinal fluid (mACSF_NaHCO3_) containing (in mM) 125 NaCl, 2.5 KCl, 1 MgCl_2_, 1.25 NaH_2_PO_4_, 2 CaCl_2_, 20 glucose, 26 NaHCO_3_, and 0.5 l-Glutamine equilibrated with 95% O_2_/5% CO_2_ and flat mounted on transparent membrane discs (Anodisc, Whatman).

### Immunohistochemistry

For tissue sections, retinas were isolated from fixed eye cups, embedded in 4% agarose and cut into 60 μm slices on a vibratome (VT1000 A, Leica). Flat-mounted retinas were cryoprotected (10% sucrose in phosphate-buffered saline (PBS) for 1 h at RT, 20% sucrose in PBS for 1 h at RT, and 30% sucrose in PBS overnight at 4 °C), frozen, and thawed three times and washed in PBS three times for 10 min at RT. Vibratome slices and flat mounts were then blocked in 5% normal donkey serum (NDS) in PBS for 1 h at RT, before being incubated with primary antibodies in 5% NDS and 0.5% Triton X-100 in PBS overnight (vibratome slices) or for 5 days (flat mounts) at 4 °C. The following primary antibodies were used in this study: mouse anti-CACNA1S to label Gpr179^38^ (1:500, Millipore, RRID:AB_2069582), sheep anti-mGluR6 (1:200, Dr. K. Martemyanov)^67^, mouse anti-CtBP2 (1:500, BD Biosciences, RRID:AB_399431), mouse anti-PKCα (1:1000, Sigma, RRID:AB_477375), rabbit anti-DsRed (1:1000, BD Biosciences, RRID:AB_394264), chicken anti-GFP (1:1000, ThermoFisher, RRID:AB_2534023). After incubation with primary antibodies, the tissue was washed in PBS three times for 10 min at RT, stained for 2 h at RT (vibratome slices) or overnight at 4 °C (flat mounts) with DyLight 405 (1:500, ThermoFisher, RRID:AB_2533208), Alexa 488 (1:1000, ThermoFisher, anti-chicken IgY, RRID:AB_2534096, anti-mouse IgG, RRID:AB_2534069), Alexa 568 (1:1000, ThermoFisher, anti-rabbit IgG, RRID:AB_143011), and Alexa 633 (1:1000, ThermoFisher, anti-mouse IgG RRID:AB_141459) secondary antibodies, washed again in PBS three times for 10 min at RT, and mounted in Vectashield medium (Vector Laboratories, RRID:AB_2336789).

### Imaging and analysis

Confocal image stacks were acquired on an Olympus Fv1000 laser scanning microscope using a 60 × 1.35 NA oil immersion objective and a 20 × 0.85 NA oil immersion objective. Dendritic and axonal connectivity of RBCs was analyzed in image stacks with 0.066–0.3 μm (*x*/*y*–*z*) voxels. Super-resolution imaging (voxel size: 0.043–0.1 μm, *x*/*y*–*z*) was performed on a Zeiss LSM 880 microscope with an AiryScan detector array. To identify dendritic synapses of individual RBCs, we generated binary masks from the signal of fluorescent proteins expressed sparsely in RBCs and the signal of immunostaining for Gpr179 or mGluR6 using local thresholding in Amira (FEI). Receptor clusters at cone synapses occur lower in the outer plexiform layer and are morphologically clearly distinct from receptor clusters at rod synapses^3, 37, 68^. Clusters of immunostaining at rods were assigned to a given RBC if the respective masks overlapped. Dendritic territories were measured as the area of the smallest convex polygon to encompass synapses in a *z*-projection. To identify ribbon release sites of individual RBCs, their axons were masked by local thresholding in Amira. Axon masks were then applied to signals of immunostaining for the C-terminal binding protein 2 in the same image stack, and synaptic clusters detected using previously described algorithms^14–16^ implemented in MATLAB (The Mathworks, RRID:SCR_001622). Axon size was measured by the surface area of the binary mask.

### Electrophysiology and visual stimulation and analysis

Cell-attached and whole-cell patch clamp recordings of ONα-RGCs were obtained from the dorsal retina in flat mount preparations^49, 69^. Throughout the recordings, retinas were continually perfused (5–7 mL/min) with warm (~33 °C) mACSF_NaHCO3_. The intracellular solution for current clamp recordings contained (in mM) 125 K-gluconate, 10 NaCl, 1 MgCl_2_, 10 EGTA, 5 HEPES, 5 ATP-Na, and 0.1 GTP-Na (pH adjusted to 7.2 with KOH). The intracellular solution for voltage clamp recordings contained (in mM) 120 Cs-gluconate, 1 CaCl_2_, 1 MgCl_2_, 10 Na-HEPES, 11 EGTA, 10 TEA-Cl, and 2 Qx314 (pH adjusted to 7.2 with CsOH). Patch pipettes had resistances of 4–7 MΩ (borosilicate glass). Signals were amplified with a Multiclamp 700B amplifier (Molecular Devices), filtered at 3 kHz (8-pole Bessel low-pass) and sampled at 10 kHz (Digidata 1440A, Molecular Devices). In voltage clamp recordings, series resistance (10–15 MΩ) was compensated electronically by ~75%. Excitatory postsynaptic currents were isolated by holding cells at the reversal potential of inhibitory (−60 mV) conductances. In current clamp recordings, no bias current was injected. ONα-RGCs were selected under infrared illumination based on their large soma size (diameter >20 μm); and correct targeting was confirmed by inclusion of Alexa 488 or Alexa 568 (0.1 mM) in the intracellular solution and 2-photon imaging at the end of each recording.

Multielectrode array (MEA) recordings were obtained from rectangular (~1 × 1.5 mm) pieces of dorsal retina and were floated RGC side down onto an MEA (Multichannelsystems, 252 electrodes, 30 μm electrode size, 100 μm center–center spacing) secured by a transparent tissue culture membrane (3 μm pore size, Corning) weighed down by a platinum ring. Retinas were continually perfused (5–7 mL/min) with warm (~33 °C) mACSF_NaHCO3_. Signals of each electrode were band-pass filtered between 300 and 3000 Hz and digitized at 10 kHz. Signal cut outs (3 ms) triggered on negative threshold crossings were written to hard disk together with the time of threshold crossing (i.e., spike time). Principal component analysis of these waveforms was used to sort spikes into trains representing the activity individual neurons (Offline Sorter, Plexon).

Visual stimuli were presented on an organic light-emitting display (OLED, eMagin) and projected onto the photoreceptor side of the retina via a substage condenser (patch clamp recordings) or through a 20 × 0.5 NA water immersion objective (MEA recordings). Photon fluxes at the preparation were calibrated with a photometer (UDT Instruments S471, 268R) and converted to photoisomerization rates based on the spectral output of the OLED measured with a Spectrometer (StellarNet, BLACK Comet), the rod spectral sensitivity, and a collecting area of 0.5 μm^2^ ^70^. Scotopic stimuli (mean intensity: 1.5 rhodopsin isomerization/rod/s, 1.5 R*) were centered on the soma of the recorded cell. To test contrast sensitivity, short luminance steps (250 ms) were presented every 2.25 s in a circular area (diameter: 300 μm)^57^. Baseline-subtracted responses (spike rate or conductance) were measured during 100 ms time windows. Spatiotemporal receptive fields were analyzed by presenting circular white noise stimuli, in which the intensity of rings of equal area centered on the recorded cell was chosen at random every 33 ms (refresh rate: 30 Hz) from a Gaussian distribution. Receptive field maps were then constructed by reverse correlation of the response with the stimulus via spike-triggered stimulus averaging^57, 58^.

### Electroretinograms

Responses to brief white light flashes (<5 ms) were acquired from *Gnat1* ^*–/–*^ and littermate control mice (P30) using a UTAS Visual Electrodiagnostic Testing System (LKC Technologies). Dark-adapted mice were anesthetized with ketamine (80 mg/kg) and xylazine (15 mg/kg) and their pupils dilated with 1% atropine sulfate (Falcon Pharmaceuticals). Recording electrodes embedded in contact lenses were placed over the cornea of both eyes. At each light level 5–10 responses were averaged. The a-wave was measured as the difference between the response minimum in the first 50 ms after flash onset and the voltage value at flash onset; and the b-wave amplitude was measured as the difference between a 15–25 Hz low-pass-filtered b-wave peak and the a-wave amplitude. ERG analysis was performed using custom scripts written in MATLAB.

### Statistics

Statistical significance of differences between morphological characteristics (e.g., territory size, average number of synapses per rod) was assessed using Wilcoxon rank sum (for two groups) or Kruskal–Wallis one-way ANOVA (for more than two groups) tests. Contrast response functions were compared using bootstrapping. The summed squared difference between mean contrast response functions of ONα-RGCs in wild-type and *Pcp2-DTA* retinas was compared to the distribution of summed squared differences generated by randomly assigning recorded contrast response functions to the two genotypes.

### Data availability

The data that support the findings of this study are available from the corresponding author upon reasonable request.

## Electronic supplementary material

Supplementary information
