# Supplementary material for: Homeostatic plasticity shapes the visual system’s first synapse
Source: Nat Commun. 2017 Oct 31;8:1220. doi: 10.1038/s41467-017-01332-7 (PMC5663853; doi:10.1038/s41467-017-01332-7)
Supplement: Supplementary file 1 — Supplementary information [file 41467_2017_1332_MOESM1_ESM.pdf]

Supplementary Figure 1

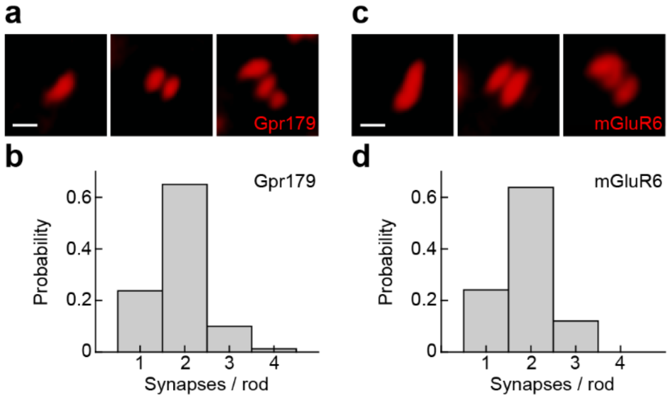

Supplementary Figure 1 | Synapse configurations visualized by super-resolution imaging. (**a**) Representative high magnification views of postsynaptic specializations in the outer plexiform layer stained for Gpr179 (red) with one (left), two (middle), or three (right) receptor clusters imaged by super-resolution microscopy (Airyscan, Zeiss). *Scale bar* represents 0.5 µm. (**b**) Summary data of the number of Gpr179 clusters per rod (n = 80 rods, n = 3 mice). (**c,d**) analogous to (**a,b**) for staining for mGluR6 (n = 83 rods, n = 2 mice).

Supplementary Figure 2

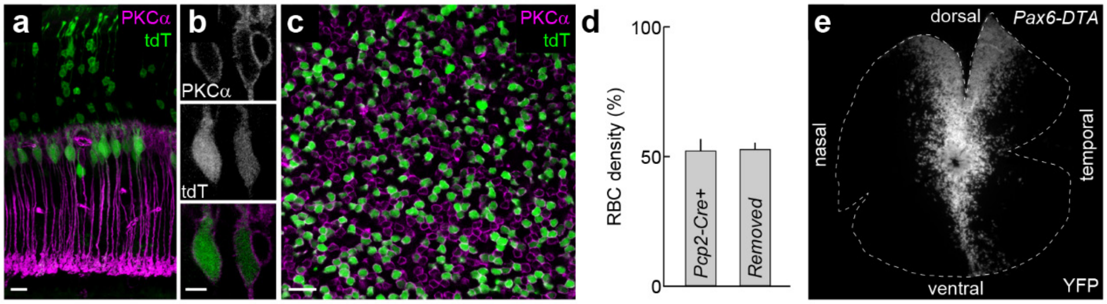

Supplementary Figure 2 | Transgenic removal of RBCs. (**a**) Crossing to a fluorescent reporter strain[1] reveals *Pcp2-Cre* expression in RBCs and a subset of photoreceptors in a retinal section. *Scale bar* represents 20 µm. (**b**) Higher magnification views of RBC somata labeled with the fluorescent reporter (tdT) and the cell-type-specific marker PKCα. *Scale bar* represents 5 µm. (**c**) Retinal whole mount shows that *Pcp2-Cre* drives recombination in approximately half of RBCs. *Scale bar* represents 20 µm. (**d**) Summary data comparing the fraction of RBCs labeled by the fluorescent reporter (n = 15 mice) and the fraction of RBCs removed in *Pcp2-DTA* mice (n = 8 mice, p > 0.7). (**e**) Flat mounted *Pax6-DTA* retina illustrating the characteristic pattern of recombination in this line, which excludes a dorsoventral wedge in the center of the retina.

Supplementary Figure 3

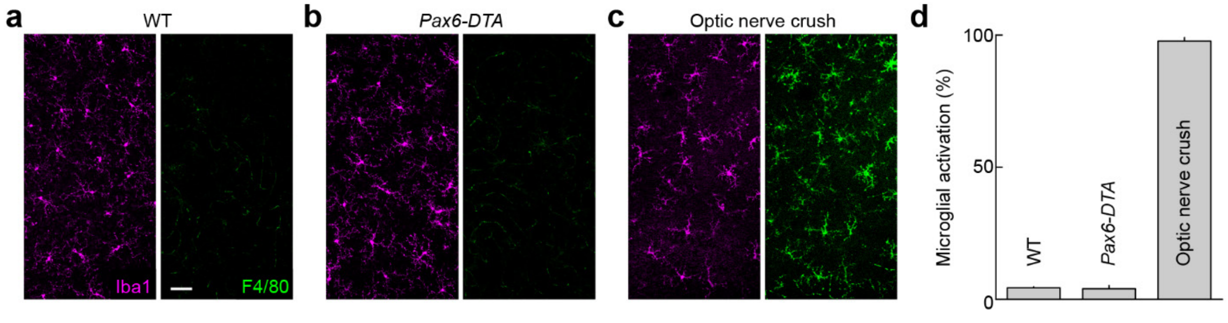

Supplementary Figure 3 | Transgenic removal of RBCs does not activate microglia. (**a-c**) Representative confocal images of retinal whole mounts in wild-type (**a**), *Pax6-DTA* (**b**), and wild-type mice two days after optic nerve crush (**c**) stained for ionized calcium binding adaptor molecule 1 (Iba1, magenta), a microglia/macrophage-specific calcium-binding protein, and F4/80 a marker of microglial activation (green). *Scale bar* represents 50 μm. (**d**) Summary data (mean ± SEM) of microglial activation in wild-type (n = 3 mice), *Pax6-DTA* (n = 4 mice), and wild-type mice after optic nerve crush (n = 2 mice).

Supplementary Figure 4

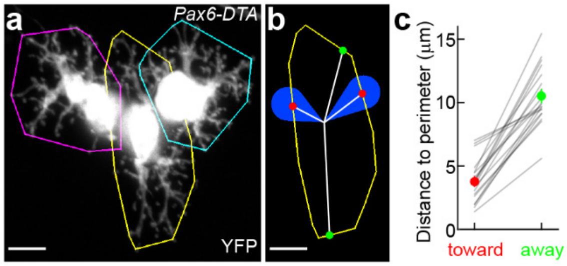

Supplementary Figure 4 | RBC dendrites grow away from remaining neighbors in *Pax6-DTA* mice. (**a**) Cluster of RBCs surrounded by RBC-depleted areas at the border of Cre-positive and Cre-negative regions of a *Pax6-DTA* retina. Outlines highlight the dendritic territories of three RBCs. *Scale bar* represents 5 µm. (**b**). Illustration of our analysis procedure for comparing dendrite growth toward and away from remaining neighbors using the middle RBC in **a** as an example. The distance from the center of its soma to the perimeter of its dendritic territory is compared for directions toward the soma centers of adjacent cells, and randomly chosen directions not hitting cell bodies of adjacent cells. *Scale bar* represents 5 µm. (**c**) Summary data show that for each RBC analyzed in this way, dendrite territories expand away from remaining neighbors ($n = 19$ RBCs, $p < 0.01$, Wilcoxon signed rank test).

Supplementary Figure 5

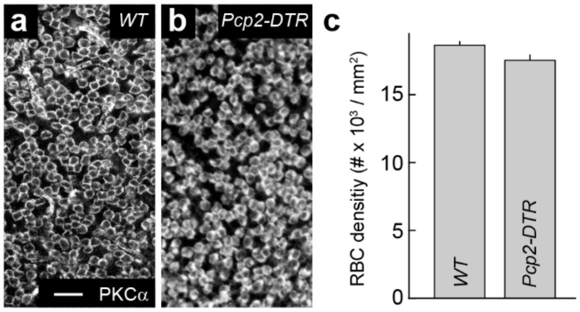

Supplementary Figure 5 | Few RBCs are removed by diphtheria toxin receptor – diphtheria toxin strategy. (**a,b**) Representative images of RBC somata stained for PKCα in retinal whole mounts of wild-type (**a**) and *Pcp2-DTR* mice (**b**) injected with diphtheria toxin (s. Methods). *Scale bar* represents 20 μm. (**c**) Summary data (mean ± SEM) comparing the RBC density in wild-type (n = 23 mice) and *Pcp2-DTR* mice injected with diphtheria toxin (n = 3 mice, $p < 0.03$).

Supplementary Figure 6

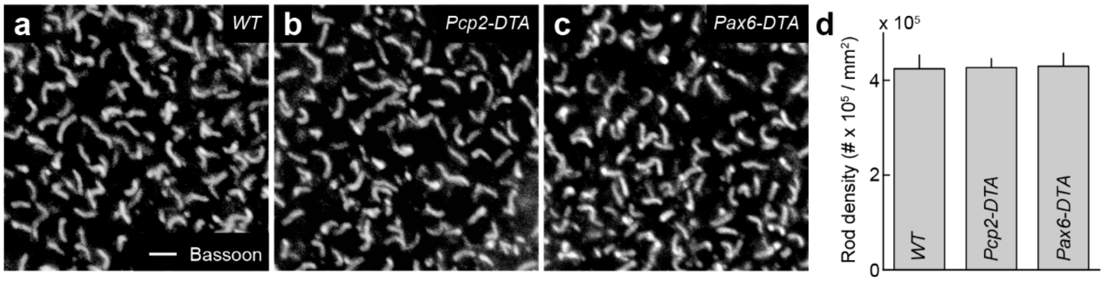

Supplementary Figure 6 | Density of rod photoreceptors is unchanged in *Pax6-DTA* and *Pcp2-DTA* mice. (**a-c**) Representative images of retinal whole mounts from wild-type (**a**), *Pcp2-DTA* (**b**), and *Pax6-DTA* mice (**c**) stained for the rod ribbon marker Bassoon. *Scale bar* represents 2 μm. (**d**) Summary data (mean ± SEM) of rod density in wild-type (n = 5 mice), *Pcp2-DTA* (n = 6 mice), and *Pax6-DTA* mice (n = 6 mice, p > 0.8 for all comparisons).

Supplementary Figure 7

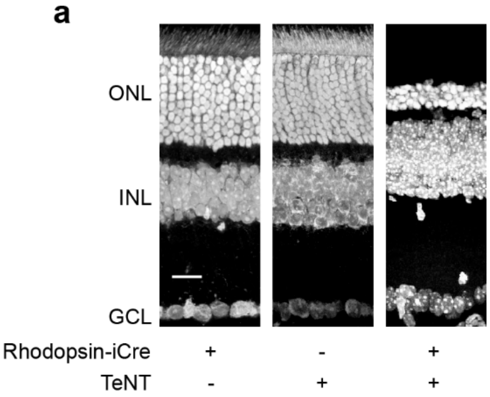

Supplementary Figure 7 | Photoreceptor degeneration in *Rhodopsin-TeNT* mice. (**a**) Representative images of vibratome sections through retinas from *Rhodopsin-iCre* mice (left), *TeNT* mice (middle), and *Rhodopsin-iCre TeNT* double positive mice (i.e. *Rhodopsin-TeNT* mice) stained with the nuclear marker Neurotrace. *Scale bar* represents 20 µm. Previous studies using a different combination of transgenic mice to express tetanus toxin in rod photoreceptors did not observe photoreceptor degeneration[2, 3]. This may be accounted for by differences in the timing and abundance of tetanus toxin expression in the different transgenic mice.

Supplementary Figure 8

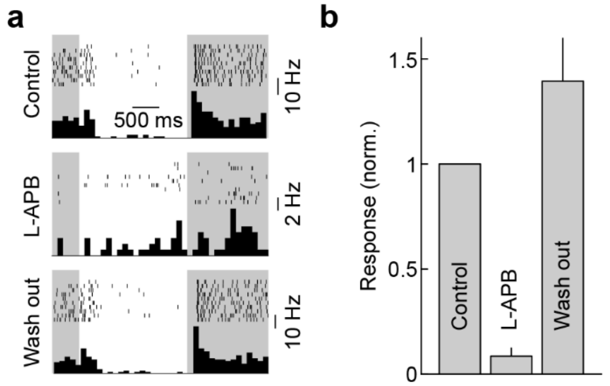

Supplementary Figure 8 | Signals at 3 R* are mediated predominantly by the rod bipolar pathway. (**a**) Representative spike rasters and histograms of an OFF-RGC responding to a dim light step (3 R*) in control conditions (top), in the presence of L-APB (50 µM, middle), and after wash out of L-APB and return to control conditions (bottom). (**b**) Summary data (mean ± SEM) normalized to firing rate in control conditions of responses of OFF-RGCs ($n = 11$). The suppression of OFF-RGC light responses ($p < 0.001$) by L-APB confirms that signals at 3 R* are mediated predominantly by the rod bipolar pathway consistent with previous findings[4].

Supplementary references

1. Madisen L*, et al.* A robust and high-throughput Cre reporting and characterization system for the whole mouse brain. *Nat Neurosci* **13**, 133-140 (2010).

2. Cao Y*, et al.* Mechanism for Selective Synaptic Wiring of Rod Photoreceptors into the Retinal Circuitry and Its Role in Vision. *Neuron* **87**, 1248-1260 (2015).

3. Wang Y*, et al.* The Auxiliary Calcium Channel Subunit alpha2delta4 Is Required for Axonal Elaboration, Synaptic Transmission, and Wiring of Rod Photoreceptors. *Neuron* **93**, 1359-1374 (2017).

4. Murphy GJ, Rieke F. Network variability limits stimulus-evoked spike timing precision in retinal ganglion cells. *Neuron* **52**, 511-524 (2006).
